# Hepatitis B virus genotypes and drug resistance mutations circulating in blood donors in Beira, Mozambique

**DOI:** 10.1371/journal.pone.0281855

**Published:** 2023-02-16

**Authors:** Ann Mathew, Nalia Ismael, Heidi Meeds, Adolfo Vubil, Ana Flora Zicai, Nédio Mabunda, Jason T. Blackard

**Affiliations:** 1 Division of Digestive Diseases, University of Cincinnati College of Medicine, Cincinnati, OH, United States of America; 2 Instituto Nacional de Saúde, Marracuene, Mozambique; CEA, FRANCE

## Abstract

Hepatitis B virus (HBV) infects nearly 300 million people and is the leading cause of hepatitis and hepatocellular carcinoma worldwide. Despite the high burden of HBV in sub-Saharan Africa, countries such as Mozambique have limited data available on circulating HBV genotypes and the presence of drug resistance mutations. Blood donors from Beira, Mozambique were tested for HBV surface antigen (HBsAg) and HBV DNA at the Instituto Nacional de Saúde in Maputo, Mozambique. Regardless of HBsAg status, donors with detectable HBV DNA were evaluated for HBV genotype. PCR was performed with primers amplifying a 2.1–2.2 kilobase fragment of the HBV genome. PCR products were submitted for next generation sequencing (NGS), and consensus sequences were evaluated for HBV genotype, recombination, and the presence or absence of drug resistance mutations. Of the 1281 blood donors tested, 74 had quantifiable HBV DNA. The polymerase gene could be amplified from 45 of 58 (77.6%) individuals with chronic HBV infection and 12 of 16 (75%) with occult HBV infection. Among these 57, 51 (89.5%) sequences belonged to HBV genotype A1, while 6 (10.5%) were HBV genotype E. All genotype E sequences were E/A recombinants, and clustered separately from other genotype E references. Genotype A samples had a median viral load of 637 IU/mL, while genotype E samples had a median viral load of 476,084 IU/mL. No drug resistance mutations were observed in the consensus sequences. The current study demonstrates the genotypic diversity of HBV in blood donors in Mozambique, but the absence of dominant (consensus) drug resistance mutations. Studies in other at-risk populations are essential for understanding the epidemiology, risk of liver disease, and likelihood of treatment resistance in resource-limited settings.

## Introduction

Despite an effective vaccine to prevent hepatitis B virus (HBV), HBV infection remains a significant public health issue. The World Health Organization estimated that approximately 296 million people were living with chronic HBV infection in 2019, and 1.5 million became newly infected each year [1]. Globally, only 10.5% of infected individuals are aware of their HBV status, and 820,000 people die each year from HBV and liver-related complications such as cirrhosis and hepatocellular carcinoma [1]. Still, HBV treatment is uncommon in resource-limited settings due to limited awareness among the general population, the cost and availability of diagnostic modalities, the cost of antiviral medications, and the lack of trained healthcare providers.

HBV is endemic in much of Africa, and significant barriers exist to reducing its associated burden of disease (reviewed in [2]). Blood testing and vaccination are commonly used prevention strategies for HBV. While guidelines to ensure blood safety often exclude potential donors that are at high risk of transfusion-transmitted infections (TTIs), HBV infections transmitted via blood transfusion remain a public health concern in many parts of the world. In Sub-Saharan Africa, blood safety is compromised by several factors including the development and implementation of national policies, the recruitment of voluntary and unpaid donors, appropriate screening of collected blood, and organizational or institutional deficits (reviewed in [3, 4]). One study reported a median risk of being infected with HBV from a blood transfusion in Sub-Saharan Africa at 4.3 infections per 1000 units and 28,595 HBV infections per year [5].

HBV is an enveloped double-stranded DNA virus with approximately 3,200 bases and four overlapping open reading frames (ORFs)–P, C, X, and S–that encode 7 proteins, including polymerase, precore, core, X proteins and 3 surface proteins (LHBs, MHBs, and SHBs). The S protein contains the “a” determinant region within the major hydrophilic region that is the target recognized by anti-HBs antibodies. Amino acid substitutions within and around the “a” determinant are associated with immune escape and vaccine failure (reviewed in [6]). We recently reported that 6.3% of >4200 full-length HBV genomes from 36 countries contained a polymorphism at a site associated with vaccine escape [7].

Genome analysis of HBV sequences demonstrate the existence of at least nine HBV genotypes that differ from one another >7.5% at the nucleotide level (reviewed in [8, 9]). The most common genotypes worldwide are C (26%), D (22%), E (18%), A (17%), and B (14%) [10]; however, the geographic distribution of these genotypes is not uniform. Genotype A is common in Europe and North America. Genotypes B and C are detected frequently in Southeast Asia and the Pacific islands. Genotype D is the most widely distributed genotype globally. Genotype E is found mainly in Africa or in individuals of African descent [11]. Genotype E infections are characterized by high e antigen positivity, elevated viral loads, and lower end of treatment response rates. Recombination between HBV genotypes also occurs leading to novel viral isolates and/or viruses with altered drug susceptibility / resistance profiles [11]. Furthermore, within a single individual, HBV–regardless of genotype–exists as a population of related, yet distinct, variants termed the viral quasispecies that facilitate rapid, adaptive changes in response to antiviral therapies, as well as immune selection pressures [12].

Like other Southern African countries, Mozambique is considered endemic for HBV [13, 14]. Population-based studies conducted in Mozambique have reported HBV infection rates of 11.4% in prisoners [15], 13.2% in refugees [16], 4.5% to 9.3% in blood donors [17, 18], 5.9% to 32.8% in persons who use drugs [19], 12.2% in young adults [13], 7.6% in HIV-positive adults initiating antiretroviral therapy [20], and 4.0% in pregnant women [21]. Most studies of HBV in Mozambique have been conducted in the capital city of Maputo, and information regarding viral diversity in the other regions of the country is scarce. In this study, next generation sequencing was utilized for the first time in Mozambique to evaluate circulating genotypes, recombination, and the presence or absence of antiviral drug resistance in HBV DNA positive blood donors from the city of Beira.

## Methods

### Study population

Between November 2014 and October 2015, a cross-sectional study was carried out at the Blood Bank of the Central Hospital in the city of Beira (population ~585,000) located in central Mozambique. The study included repository and voluntary blood donors. All study participants (n = 1281) provided written consent and demographic information according to a structured questionnaire. From each participant, 9 mL was collected in vacutainers with K3EDTA (Becton Dickinson, Franklin Lakes, NJ, USA). This study was approved by the National Bioethics Committee in Mozambique (number 263/CNBS/2014).

### Serological assays and DNA detection / quantification

Plasma was obtained from whole blood for further testing. Screening for HBV surface antigen (HBsAg) was performed at the Blood Bank using a commercially available Advanced Quality HBsAg ELISA Test Kit (InTec Products, INC, China), and those with reactive results were subsequently confirmed with a rapid test–Advanced Quality HBsAg Rapid Test (InTec Products, INC, China). HBV DNA quantification was performed at the Instituto Nacional de Saúde using the COBAS AmpliPrep/COBAS TaqMan HBV Test, v2.0 (Roche Diagnostics, Germany) with a detection limit of 20 IU/mL according to the manufacturer’s instructions. HIV testing and viral load quantification were performed as described previously [18].

### HBV amplification and sequencing

Viral DNA was extracted from 200 uL of plasma using QIAamp UltraSens Virus Kit (Qiagen, Germantown, Maryland, USA) with a final elution volume of 60 uL in dH_2_O. Extracted HBV DNA was then used to amplify the S and P region of the viral genome using one of three primers sets–A, B, and C. Primer set A consisted of the forward primer 5’–GTG TGG ATT CGC ACT CCT– 3’ (position 2269–2287 relative to the EcoRI site) and the reverse primer 5’–CCG ATG AGC TTT GCT CCA GAC C– 3’ (position 1328–1307). Primer set B used the same forward primer as set A and the reverse primer 5’–CGT CAG CAA ACA CTT GGC– 3’ (position 2269–2287), while primer set C included the forward primer 5’–GGG CAG GTC CCC TAG AAG AAC T– 3’ (position 2363–2386) and the reverse primer from set A. Primer set B was used initially for all samples. For any samples that were PCR negative, primer sets A or C were then used for a second PCR attempt. The PCR Reaction Mixture was prepared to a final reaction volume of 50 uL using 25 uL of PicoMaxx 2x master mix, 2 uL each of 10uM forward and reverse primers, 19 uL of dH_2_O, and 2 uL of template DNA. The thermocycler was programmed for a total of 40 cycles with an initial denaturation at 95°C for 2 minutes, 94°C for 40 seconds, 60°C for 90 seconds, and 68°C for 3 minutes, and a final extension at 68°C for 8 minutes. The amplified PCR products were then subjected to gel electrophoresis on a 1% agarose gel, and PCR bands between 2.1–2.3 kilobases were isolated using the QIAEX II Gel Extraction Kit (Qiagen, Germantown, Maryland, USA).

### Next generation sequencing

PCR products were sent to the University of Cincinnati College of Medicine Genomics, Epigenomics and Sequencing Core for next generation sequencing (NGS). Library preparation was performed using the NEBNext Ultra II FS DNA library prep kit and sequenced on an Illumina HiSeq 1000 sequencer with the setting SR 1 x 51 base pairs. Reads generated were run through FastQC for quality control, and no reads were flagged as poor quality. All tools utilized were run under default parameters. Reads for each sample were then mapped to a reference genome–AY233282 from South Africa–in UGENE version 39.0 [22] to generate a consensus.

### Phylogenetic analysis

Nucleotide alignments were performed with Clustal X 2.1 [23], and additional phylogenetic inference was performed using a Bayesian Markov Chain Monte Carlo (MCMC) approach as implemented in the Bayesian Evolutionary Analysis by Sampling Trees (BEAST) version 1.10.4 program [24] with an uncorrelated log-normal relaxed molecular clock, general time-reversible model, and nucleotide site heterogeneity estimated using a gamma distribution. The MCMC analysis was run for a chain length of 1,000,000,000. and results were visualized to confirm adequate chain convergence with Tracer version 1.7.2. The effective sample size (ESS) was calculated for each parameter, and all ESS values were >2000 indicating sufficient sampling. The maximum clade credibility tree was selected from the posterior tree distribution after a 10% burn-in using Tree Annotator version 1.10.4 and visualized in FigTree version 1.4.4 as described previously [25, 26].

### Recombination analysis

The jumping profile Hidden Markov Model (jpHMM) program was utilized to detect potential intergenotypic recombination [27]. Putative recombinants were then confirmed using SimPlot version 3.5.1 [28] with a 300 base pair (bp) window, 30 bp step, and 1,000 replicates. Diversity plots were initially performed with one representative sequence each for genotypes A1, A2, A3, A5, A6, B, C, D1-D7, D10, E, F, G, and H. Bootscan analysis was then performed with two non-recombinant “parents”–AY233282 (genotype A1) from South Africa and JQ000009 (genotype E) from Argentina, as well as AB059659 (genotype H) from the United States as an outlier.

### Data availability

The raw NGS reads are available in Bioproject PRJNA851915 as SRR19789133 –SRR19789152 and SRR19880356 –SRR19880392. Consensus HBV sequences are available in GenBank under the accession numbers ON854557 –ON854613.

### Drug resistance mutation analysis and vaccine escape mutation analysis

The polymerase open reading frame (P ORF) was translated to amino acids using the Babylon Translator tool from the Hepatitis Virus Diversity Research database to detect drug resistance mutations among the samples [29]. The translated P ORF was visualized in AliView [30]. The “HBV RT: Mutation prevalence according to genotype and treatment” tool from the Stanford HBVSeq database [31, 32] and other resistance mutations reported in literature were analyzed for drug resistance mutations in AliView. Similarly, vaccine escape mutations were analyzed by translating the Surface ORF and visually inspecting the ‘a’ determinant at sites T116, P120, T126, Q129, M133, F134, K141, P142, D144, and G145 within AliView and presented as a WebLogo [33].

## Results

From the 1281 samples, 74 (5.8%) had quantifiable HBV DNA. Forty-five of 58 (77.6%) individuals with chronic HBV and 12 of 16 (75%) with occult HBV infection could be amplified using an in-house assay for the polymerase ORF. Fifty-four (94.7%) of the blood donors were male (Table 1). Twenty-eight (49.1%) donors were in the 18–24 age range, 46 (80.7%) were single, and 50 (87.7%) had completed secondary education. Thirty-six (63.2%) individuals had HBV DNA levels <2000 IU/mL (<3.30 log_10_ IU/mL). The median HBV DNA level was 3.11 log_10_ IU/mL (range: 1.00 to 8.23) for individuals with chronic HBV infection compared to 2.59 (range: 1.00 to 5.99) for individuals with occult HBV infection. This difference in median HBV levels between genotype A and genotype E/A samples was statistically significant (p = 0.0063). Two individuals were HIV positive, including BSB0014 with an HIV viral load of 4,075 copies/mL and BSB476 with an HIV viral load of 80,170 copies/mL.

**Table 1:** Sociodemographic characteristics of the 57 study participants.

Characteristic	Number (Percent)
Gender	
Male	54 (94.7)
Female	3 (5.3)
Age (years)	
<18	2 (3.5)
18–24	28 (49.1)
25–34	18 (31.6)
35–44	7 (12.3)
45–65	2 (3.5)
Education	
Primary	6 (10.5)
Secondary	50 (87.7)
Higher	1 (1.8)
Donation type	
Regular	27 (47.4)
Replacement	30 (52.6)
Marital Status	
Single	46 (80.7)
Married	11 (19.3)
HBV viral load (IU/mL)	
<2,000	36 (63.2)
2,000–20,000	11 (19.3)
>20,000	10 (17.5)
Type of HBV infection	
Chronic	45 (78.9)
Occult	12 (21.1)

**Fig 1:**
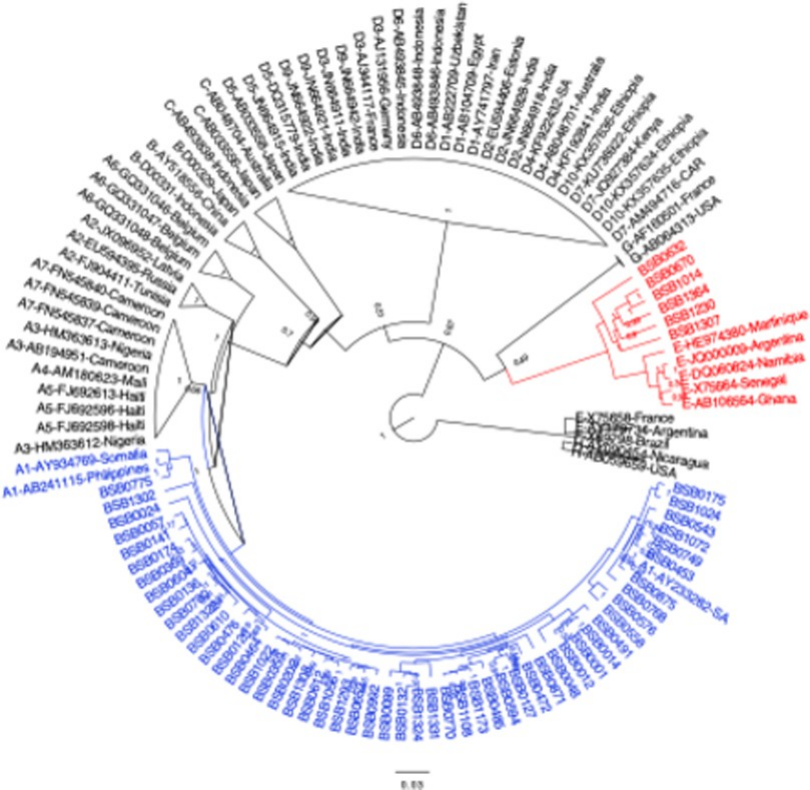
Phylogenetic analysis of 57 partial HBV genomes from Beira, Mozambique. Genotype A sequences from Mozambique are shown in blue, while genotype E/A recombinant sequences are highlighted in red. GenBank reference sequences are indicated by their genotype/subtype, accession number, and country of origin. Relevant posterior probabilities >0.90 out of 1.00 are shown. The scale bar indicates 0.03 nucleotide substitutions per site.

**Fig 2:**
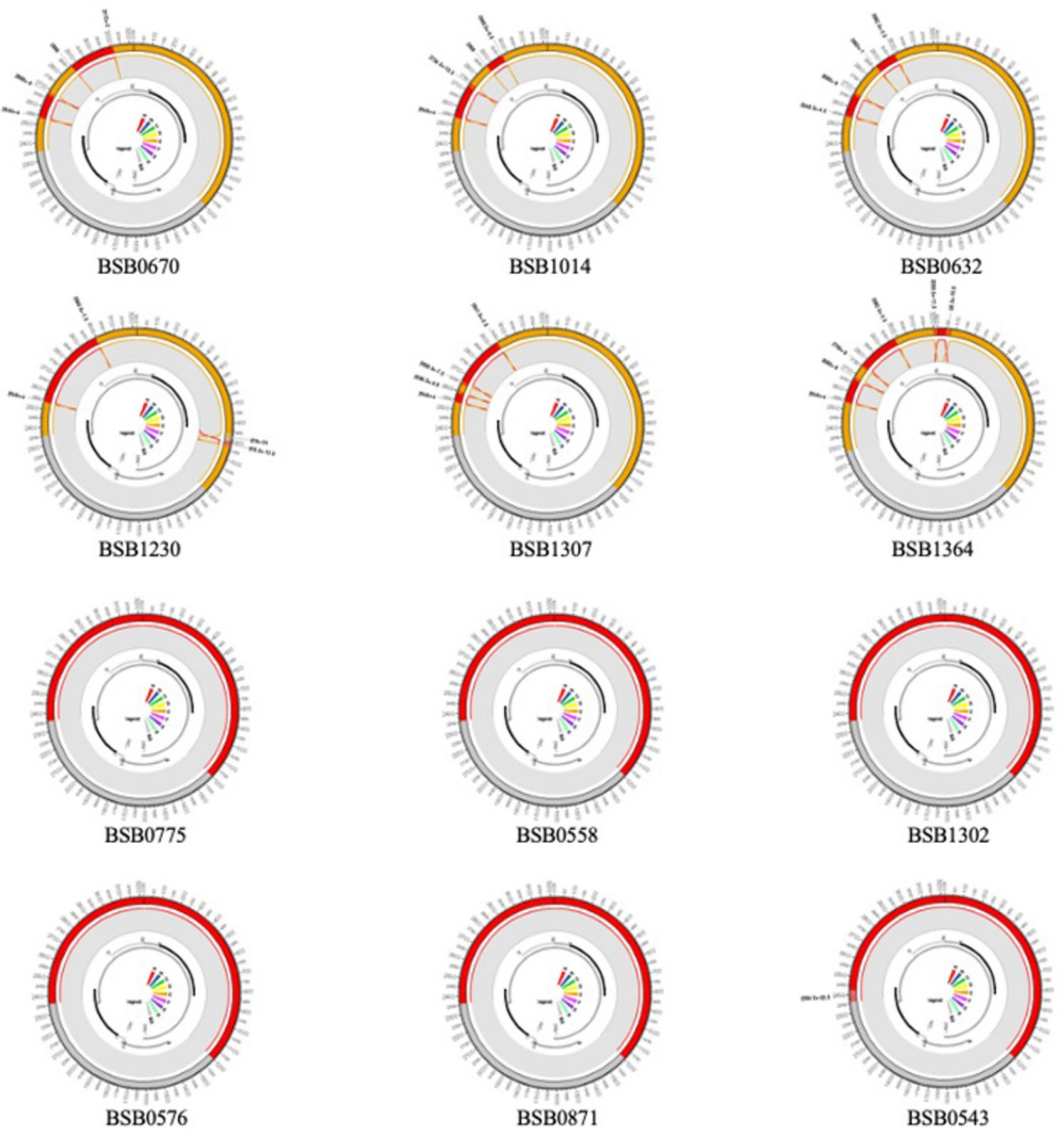
Recombination analysis for **(A)** genotype E/A recombinant viruses and **(B)** representative non-recombinant genotype A viruses.

NGS analysis produced an average of 1,063,988 reads per sample. Sequences had an average length of 2,208 bases (S1 Table). By phylogenetic analysis, 51 (89.5%) sequences belonged HBV to genotype A1, while 6 (10.5%) belonged to HBV genotype E (Fig 1). Because the genotype E sequences from Mozambique clustered separately from other genotype E references, the potential for intergenotypic recombination was evaluated. As shown in Fig 2A, all genotype E sequences–BSB0670, BSB1014, BSB0632, BSB1230, BSB1307, and BSB1364 –were recombinants between genotypes E and A. No non-recombinant genotype E sequences were observed. In contrast, all genotype A sequences from Mozambique were non-recombinant viruses (data shown for 6 representative genotype A sequences in Fig 2B). While recombination events were largely localized to the polymerase ORF, the distinct recombination events (Fig 2) and relatively long branch lengths (Fig 1) suggest the presence of multiple E/A recombinant viruses circulating in Mozambique. Recombination was confirmed by bootscan analysis as shown in S1 Fig.

**Fig 3:**
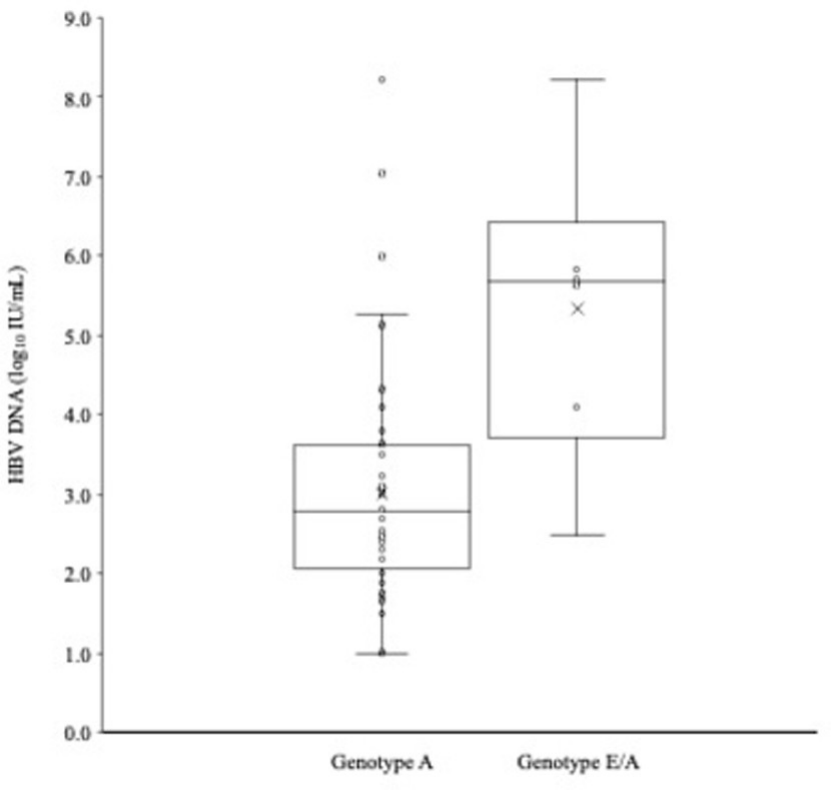
HBV viral loads (log_10_ IU/mL) for genotype A samples compared to genotype E/A recombinant samples.

As shown in Fig 3, HBV genotype A viruses had a median viral load of 2.77 log_10_ IU/mL (range: 1.00 to 8.23), while HBV recombinant E/A viruses had a median viral load of 5.68 log_10_ IU/mL (range: 2.49 to 8.23).

No drug resistance mutations were observed in the polymerase ORF of any sequence from Mozambique. However, amino acid polymorphisms within the reverse transcriptase (rt) domain of the P ORF were observed for all samples except BSB1014 (chronic; E/A recombinant) and BSB0632 (chronic; E/A recombinant). Polymorphism at position rtD7 was detected among all genotype A samples. Similarly, polymorphisms at positions rtI53, rtS109, rtN122, rtN124, rtM129, rtN131D, and rtV163 were frequently identified among most genotype A samples (Table 2).

**Table 2:** Amino acid polymorphisms within the reverse transcriptase (rt) domain of the polymerase open reading frame were observed in all samples except BSB1014 (genotype E/A) and BSB0632 (genotype E/A). The polymorphism at rtD7 was detected in all genotype A samples. Similarly, polymorphisms at rtI53, rtS109, rtN122, rtN124, rtM129, rtN131D, and rtV163 were frequently identified among genotype A samples.

Patient ID	HBV status	HBV genotype	Mutations
**BSB0001**	Chronic	A	rtD7V, rtI53L, rtS109P, rtN122H, rtY126H, rtM129L, rtN131D, rtV163I
**BSB0012**	Chronic	A	rtD7V, rtI53L, rtS109P, rtM129L, rtN131D, rtV163I
**BSB0014**	Occult	A	rtD7V, rtI53L, rts109P, rtN122H, rtY126H, rtN131D, rtV163I
**BSB0024**	Chronic	A	rtD7V, rtI53L, rtS109P, rtN122H, rtN124H, rt126H, rtM129L, rtN131D, rtV163I, rtR18K
**BSB0048**	Chronic	A	rtD7V, rtI53L, rtS109P, rtN131D, rtV163I, rtR110G, rtW153R
**BSB0057**	Occult	A	rtD7I, rtI53P, rtS109P, rtM129L, rtN131D, rtV163I, rtW153R
**BSB0099**	Chronic	A	rtD7V, rtI53L, rtS109P, rtN122H, rtY126H, rtM129L, rtN131D, rtV163I, rtM145L, rtN131D
**BSB0126**	Chronic	A	rtD7V, rtI53P, rtS109P, rtN122H, rtN124H, rtM129L, rtV163I, rtN118D
**BSB0127**	Chronic	A	rtD7V, rtI53L, rtS109P, rtN122H, rtY126H, rtM129L, rtV163I
**BSB0132**	Chronic	A	rtD7V, rt53L, rtS109P, rtN122H, rtY126H, rtM129L, rtN131D, rtV163I
**BSB0136**	Chronic	A	rtD7V, rtI53P, rtS109P, rtN122H, rtN124H, rtM129L, rtN131D, rtV163I
**BSB0141**	Occult	A	rtD7V, rtI53L, rtS109P, rtN122H, rtN124H, rtM129L, rtN131D, rtV163I
**BSB0174**	Chronic	A	rtD7V, rtI53P, rtS109P, rtN122H, rtN124H, rtM129L, rtN131D, rtV163I
**BSB0175**	Chronic	A	rtD7V, rtI53L, rtS109P, rtN122H, rtM129L, rtV163I, rtR110G
**BSB0202**	Chronic	A	rtD7V, rtI53L, rtS109P, rtN122H, rtN124H, rtM129L, rtN131D, rtV163I
**BSB0359**	Chronic	A	rtD7V, rtI53L, rtN122H, rtN124H, rtM129L, rtN131D, rtV163I, rtH13N
**BSB0369**	Chronic	A	rtD7V, rtI53P, rtN122H, rtN124H, rtM129L, rtN131D, rtV163I
**BSB0453**	Chronic	A	rtD7V, rtI53L, rtS109P, rtN122H, rtH126Y, rtM129L, rtV163I, rtH13Y, rtR110G
**BSB0464**	Chronic	A	rtD7V, rtI53L, rtS109P, rtN122H, rtN124H, rtM129L, rtN131D, rtV163I, rtW153R
**BSB0472**	Chronic	A	rtD7V, rtI53P, rtS109P, rtN122H, rtM129L, rtN131D, rtV163I, rtT54N, rtS57P
**BSB0476**	Chronic	A	rtD7V, rtI53L, rtS109P, rtN122H, rtN124H, rtM129L, rtN131D, rtV163I
**BSB0485**	Chronic	A	rtD7V, rtI53L, rtS109P, rtN122H, rtM129L, rtN131D, rtV163I, rtE1D, rtD2N
**BSB0491**	Chronic	A	rtD7V, rtI53L, rtS109P, rtN122H, rtM129L, rtN131D, rtV163I, rtN76K
**BSB0543**	Chronic	A	rtD7V, rtI53L, rtS109P, rtN122H, rtM129L, rtV163I, rtI103V, rtR110G
**BSB0558**	Chronic	A	rtD7V, rtI53L, rtS109P, rtN122H, rtH126Y, rtM129L, rtN131D, rtP161A, rtV163I, rtI103V, rtR110G, rtW153R
**BSB0576**	Occult	A	rtD7V, rtI53L, rtS109P, rtN122H, rtH126Y, rtM129L, rtV163I, rtI103V, rtW153R
**BSB0594**	Chronic	A	rtD7V, rtI53L, rtS109P, rtN122H, rtN131D, rtV163I, rtM129L, rtY126H, rtS57P
**BSB0604**	Chronic	A	rtD7V, rtI53L, rtS109P, rtN122H, rtN124H, rtM129L, rtN131D, rtV163I, rtR18K, rtR110G, rtV112I, rtV142G
**BSB0610**	Occult	A	rtD7V, rtI53L, rtS109P, rtN122H, rtN124H, rtM129L, rtN131D, rtV163I
**BSB0612**	Occult	A	rtD7V, rtI53L, rtS109P, rtN122H, rtN124H, rtM129L, rtN131D, rtV163I
**BSB0632**	Chronic	E/A	
**BSB0652**	Chronic	A	rtD7V, rtI53L, rtS109P, rtN122H, rtN124H, rtM129L, rtN131D, rtV163I, rtL164M, rtI16T, rtK149Q, rtW153R, rtL157M
**BSB0670**	Chronic	E/A	rtY111C, rtM145L
**BSB0749**	Occult	A	rtD7V, rtI53P, rtS109P, rtV112A, rtN122H, rtN126Y, rtM129L, rtV163I
**BSB0768**	Occult	A	rtD7V, rtI53L, rtS109P, rtN122H, rtY126H, rtM129L, rtN131D, rtV163I, rtV112L, rtL140I
**BSB0770**	Occult	A	rtD7V, rtI53L, rtS109P, rtN122H, rtM129L, rtN131D, rtV163I, rtR110G
**BSB0775**	Chronic	A	rtD7A, rtN122H, rtH126Y, rtM129L, rtV163I
**BSB0780**	Chronic	A	rtD7V, rtI53P, rtS109P, rtN122H, rtN124H, rtM129L, rtN131D, rtV163I, rtI16T
**BSB0871**	Chronic	A	rtD7V, rtI53V, rtS75T, rtS109P, rtV112A, rtN122H, rtM129L, rtN131D, rtV163I, rtQ139E
**BSB0875**	Chronic	A	rtD7V, rtI53P, rtS109P, rtN122H, rtH126Y, rtM129L, rtV163I
**BSB0992**	Chronic	A	rtD7V, rtI53L, rtS109P, rtN122H, rtN124H, rtM129L, rtN131D, rtV163I
**BSB1014**	Chronic	E/A	
**BSB1022**	Chronic	A	rtD7V, rtI53L, rtS109P, rtN122H, rtN124H, rtN131D, rtV163I
**BSB1024**	Occult	A	rtD7V, rtI53L, rtS109P, rtN122H, rtM129C, rtV163I, rtI91L
**BSB1056**	Chronic	A	rtD7V, rtI53L, rtS109P, rtN122H, rtN124H rtM129L, rtN131D, rtV163I
**BSB1072**	Chronic	A	rtD7V, rtI53L, rtS109P, rtN122H, rtM129L, rtV163I, rtI91L
**BSB1108**	Chronic	A	rtD7V, rtI53L, rtS109P, rtN122H, rtM129L, rtN131D, rtV163I
**BSB1173**	Chronic	A	rtD7V, rtI53P, rtS109P, rtN122H, rtM129L, rtN131D, rtV163I
**BSB1230**	Chronic	E/A	rtK11R
**BSB1293**	Occult	A	rtD7T, rtI53L, rtN122H, rtN124H, rtM129L, rtN131D, rtV163I, rtI103V
**BSB1302**	Chronic	A	rtD7A, rtN122H, rtM129L, rtV163I, rtQ139E
**BSB1307**	Chronic	E/A	rtK11R, rtP130S
**BSB1308**	Chronic	A	rtD7V, rtI53L, rtS109Q, rtN122H, rtN124H, rtM129L, rtN131D, rtV163I
**BSB1322**	Chronic	A	rtD7V, rtI53L, rtS109P, rtN122H, rtN124H, rtM129L, rtN131D
**BSB1324**	Chronic	A	rtD7V, rtI53L, rtS109P, rtN122H, rtY126H, rtM129L, rtN131D, rtV163I, rtM145L
**BSB1331**	Chronic	A	rtD7V, rtI53L, rtS109P, rtN122H, rtM129L, rtN131D, rtW153R, rtI16T
**BSB1364**	Occult	E/A	rtL91I

**Fig 4:**
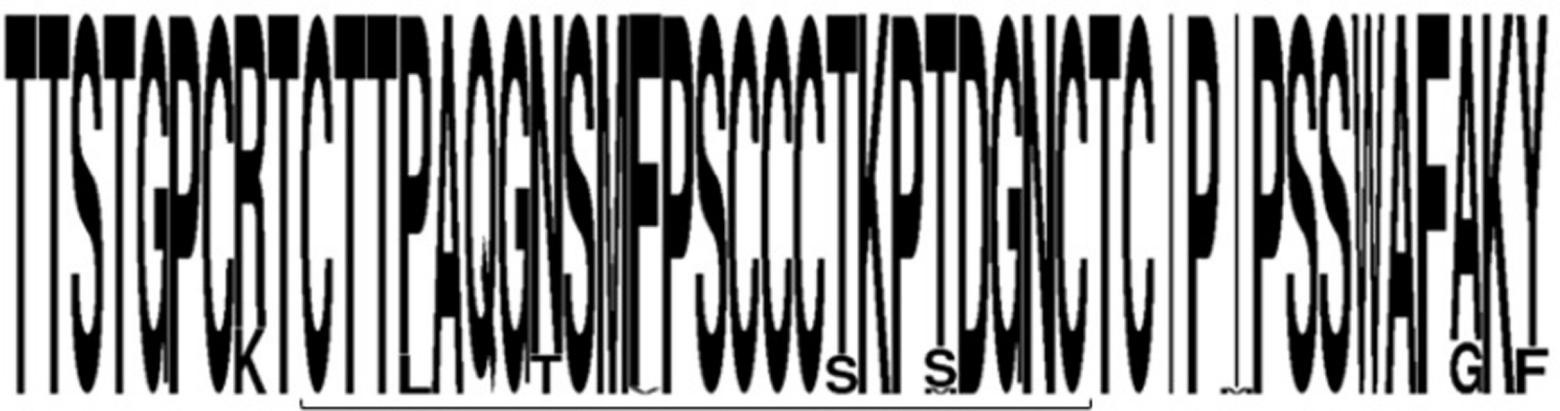
Variation within the “a” determinant (denoted by bracket) of consensus HBV surface gene sequences shown as a frequency plot.

Within the “a’’ determinant region of the Surface ORF, polymorphisms were noted at positions P127 (L in 6 genotype E/A sequences), N131 (T in 6 genotype E/A sequences), F134 (V in 1 genotype A sequence), T140 (S in 6 genotype E/A sequences), and T143 (S in 6 genotype E/A sequences, M in 1 genotype A sequence); however, no previously identified vaccine escape mutations were identified (Fig 4).

## Discussion

Population-based studies conducted in Mozambique have reported HBV infection rates of 11.4% in prisoners [15], 13.2% in refugees [16], 4.5% to 9.3% in blood donors [17, 18], 5.9% to 32.8% in persons who use drugs [19], 12.2% in young adults [13], 7.6% in HIV-positive adults initiating antiretroviral therapy [20], and 4.0% in pregnant women [21].

Previous studies suggest that genotypes A and E are circulating in various at-risk populations in Mozambique [20, 34, 35]. Chambal *et al*. evaluated HBV genotypes circulating in antiretroviral therapy (ART) naïve persons with HIV/HBV co-infection in Maputo using the TRUGENE HBV Genotyping Kit Module 2.0 to amplify a ~1.2 kilobase fragment of the viral genome [34]. Genotype A was identified in 25 of 27 patients and genotype E in 2 patients. Mabunda *et al*. evaluated HBV genotypes in blood donors with occult HBV infection in Maputo by amplifying a ~900 base portion of the S/P ORF [35]. Eight patients had genotype A1 infections, while 1 had a genotype E infection. Wandeler *et al*. enrolled HIV-positive adults initiating ART in Cabo Delgado (northern Mozambique) and amplified a ~1000 base portion of the S/P ORF [20]. Genotype A was detected most frequently, along with genotype E. Cunha *et al*. utilized a line probe assay to detect genotypes A (86.3%), D (0.5%), E (8.5%), and dual infections (4.7%) in blood donors [17]. The present study also revealed that subgenotype A1 is the predominant genotype circulating in Beira, Mozambique.

Interestingly, this is the first study to identify E/A recombinant viruses in Mozambique. Intergenotypic recombinant genomes–including E/A recombinants–have been reported in several West and Central African countries [25, 26, 36–42]. Thus, the occurrence of E/A recombinant viruses in Mozambique may reflect migration between Mozambique and other regions of Africa. Moreover, recombination may facilitate the emergence of novel viral genomes with higher replication capacity as noted by the higher HBV DNA levels observed here for E/A recombinant viruses compared to non-recombinant genotype A viruses. Thus, full-length genome analysis is underway to determine if genotype A sequences–based on partial genome sequencing–may contain recombination events in the non-sequenced regions and to further characterize the novel recombinant viruses that were identified here.

The prevalence of occult HBV infection is largely unknown in Mozambique. A cross-sectional study of ART-naïve HIV-positive individuals in Maputo found that 17 of 206 (8.3%) individuals with isolated anti-core antibodies had detectable HBV DNA indicative of occult HBV infection [43]. In our study of HBsAg-negative blood donors, 0.98% had occult HBV infection [35]. Occult HBV has been reported in other African countries with a regional prevalence of 26.5% in the South, 11% in the North, 9.1% in the East, and 8.5% in the West [44]. To our knowledge, HBV drug resistance has been evaluated in Mozambique in two studies. Chambal *et al*. observed no 3TC/lamivudine resistance mutations in ART-naïve persons with HIV/HBV co-infection [34]. Wandeler *et al*. reported drug resistance or limited in 5 of 102 HIV-positive adults initiating ART [20]. Although no drug resistance mutations were observed in the current study, amino acid polymorphisms were present in multiple sequences. Viral diversity is shaped by several factors including the error rate of the viral polymerase, the production rate of new virions, immune-mediated selection pressures, and the presence or absence of antiviral selection pressure. Given the modest sample size and presence of distinct genotypes–non-recombinant A and recombinant A/E–the current study was not powered to evaluate the clinical significance of these polymorphisms. Larger cohort-based studies, as well as *in vitro* characterization of polymerase gene polymorphisms, are needed.

While HBV treatment in mono-infected persons is not routine in Mozambique, a study from neighboring South Africa detected HBV lamivudine-resistance in 3 of 15 treatment-naïve individuals with chronic HBV infection and in 10 of 20 HBV/HIV co-infected individuals [45], suggesting that drug resistance may occur even in untreated individuals. Infrequent screening, limited supply of antiviral drugs, and poor access to clinical monitoring contribute to the emergence of drug resistance in Africa and highlight the need for continued monitoring in key populations [46]. Finally, we and others have evaluated the global presence of vaccine escape mutations [7, 46, 47]. Polymorphisms at sites associated with vaccine escape is relatively common; however, it is often not possible to determine if the individuals harboring these polymorphisms or vaccine-escape mutations had been vaccinated against HBV.

Collectively, our data suggest that genotype A and E/A recombinant viruses are common in blood donors in Beira, Mozambique and that drug resistance is rare.

## Supporting information

S1 Fig Bootscan analysis for A) BSB0632, B) BSB0670, C) BSB1014, D) BSB1230, E) BSB1307, and F) BSB1364 using SimPlot version 3.5.1 with a window of 300, step of 30, and 1,000 replicates. References include AY233282 (genotype A1 from South Africa; blue line), JQ000009 (genotype E from Argentina, red line), and AB059659 (genotype H from the United States, black line).
(ZIP)

S1 Table PCR positive samples had an average length of 2208 nucleotides and produced an average of 1,063,988 reads. (DOCX)

S1 Questionnaire Inclusivity questionnaire. (DOCX)
