# Supplementary figures and images for: Hepatitis B virus genotypes and drug resistance mutations circulating in blood donors in Beira, Mozambique
Source: PLoS One. 2023 Feb 16;18(2):e0281855. doi: 10.1371/journal.pone.0281855 (PMC9934330; doi:10.1371/journal.pone.0281855)

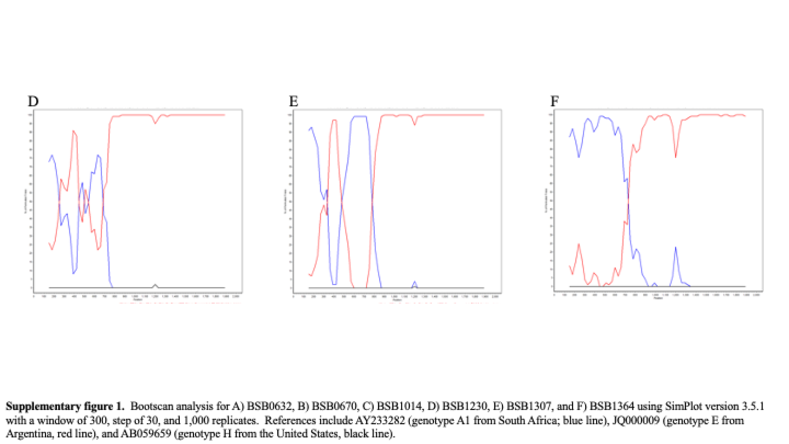

Supplement: S1 Fig — Bootscan analysis for A) BSB0632, B) BSB0670, C) BSB1014, D) BSB1230, E) BSB1307, and F) BSB1364 using SimPlot version 3.5.1 with a window of 300, step of 30, and 1,000 replicates. References include AY233282 (genotype A1 from South Africa; blue line), JQ000009 (genotype E from Argentina, red line), and AB059659 (genotype H from the United States, black line). (ZIP) [file pone.0281855.s001.zip › S1_Figabc.tif]

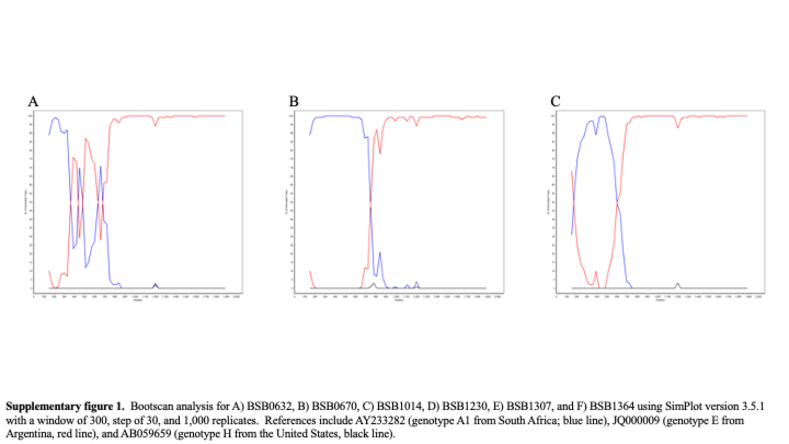

Supplement: S1 Fig — Bootscan analysis for A) BSB0632, B) BSB0670, C) BSB1014, D) BSB1230, E) BSB1307, and F) BSB1364 using SimPlot version 3.5.1 with a window of 300, step of 30, and 1,000 replicates. References include AY233282 (genotype A1 from South Africa; blue line), JQ000009 (genotype E from Argentina, red line), and AB059659 (genotype H from the United States, black line). (ZIP) [file pone.0281855.s001.zip › S1_Figdef.tif]
