# Supplementary material for: Hepatitis B virus genotypes and drug resistance mutations circulating in blood donors in Beira, Mozambique
Source: PLoS One. 2023 Feb 16;18(2):e0281855. doi: 10.1371/journal.pone.0281855 (PMC9934330; doi:10.1371/journal.pone.0281855)
Supplement: S1 Table — (DOCX) [file pone.0281855.s002.docx]

**Supplementary table 1.** PCR positive samples had an average length of 2208 nucleotides and produced an average of 1,063,988 reads.

| Patient ID | HBV status | HBV genotype | Number of reads | Length of consensus |
| --- | --- | --- | --- | --- |
| BSB0001 | Chronic | A | 950 | 2284 |
| BSB0012 | Chronic | A | 1,907,365 | 2179 |
| BSB0014^1^ | Occult | A | 258,023 | 2296 |
| BSB0024 | Chronic | A | 96 | 2216 |
| BSB0048 | Chronic | A | 15,564 | 2284 |
| BSB0057 | Occult | A | 1,433,836 | 2172 |
| BSB0099 | Chronic | A | 160 | 2254 |
| BSB0126 | Chronic | A | 1,168,687 | 2172 |
| BSB0127 | Chronic | A | 154 | 2192 |
| BSB0132 | Chronic | A | 150 | 2192 |
| BSB0136 | Chronic | A | 1,415,494 | 2169 |
| BSB0141 | Occult | A | 842,451 | 2168 |
| BSB0174 | Chronic | A | 1,674,198 | 2171 |
| BSB0175 | Chronic | A | 479,345 | 2226 |
| BSB0202 | Chronic | A | 727,460 | 2176 |
| BSB0359 | Chronic | A | 1,493,036 | 2174 |
| BSB0369 | Chronic | A | 1,028,378 | 2169 |
| BSB0453 | Chronic | A | 1,253,618 | 2180 |
| BSB0464 | Chronic | A | 485,960 | 2167 |
| BSB0472 | Chronic | A | 1,395,231 | 2142 |
| BSB0476^2^ | Chronic | A | 1,178,743 | 2605 |
| BSB0485 | Chronic | A | 1,708,833 | 2172 |
| BSB0491 | Chronic | A | 1,738,555 | 2177 |
| BSB0543 | Chronic | A | 575,064 | 2269 |
| BSB0558 | Chronic | A | 1,435,673 | 2172 |
| BSB0576 | Occult | A | 1,464,053 | 2171 |
| BSB0594 | Chronic | A | 897,214 | 2287 |
| BSB0604 | Chronic | A | 1,473,859 | 2179 |
| BSB0610 | Occult | A | 1,440,754 | 2174 |
| BSB0612 | Occult | A | 1,317,294 | 2169 |
| BSB0632 | Chronic | E/A | 884,711 | 2169 |
| BSB0652 | Chronic | A | 1,322,674 | 2173 |
| BSB0670 | Chronic | E/A | 116,730 | 2291 |
| BSB0749 | Occult | A | 1,618,919 | 2168 |
| BSB0768 | Occult | A | 88 | 2175 |
| BSB0770 | Occult | A | 1,064,089 | 2173 |
| BSB0775 | Chronic | A | 1,389,049 | 2175 |
| BSB0780 | Chronic | A | 1,942,782 | 2174 |
| BSB0871 | Chronic | A | 1,740,135 | 2163 |
| BSB0875 | Chronic | A | 1,734,435 | 2179 |
| BSB0992 | Chronic | A | 1,373,716 | 2176 |
| BSB1014 | Chronic | E/A | 786,060 | 2557 |
| BSB1022 | Chronic | A | 114694 | 2177 |
| BSB1024 | Occult | A | 787,709 | 2315 |
| BSB1056 | Chronic | A | 1,449,972 | 2175 |
| BSB1072 | Chronic | A | 840,082 | 2255 |
| BSB1108 | Chronic | A | 2,060,879 | 2171 |
| BSB1173 | Chronic | A | 1,636,850 | 2175 |
| BSB1230 | Chronic | E/A | 785,795 | 2163 |
| BSB1293 | Occult | A | 1,654,946 | 2180 |
| BSB1302 | Chronic | A | 1,017,496 | 2177 |
| BSB1307 | Chronic | E/A | 711,449 | 2164 |
| BSB1308 | Chronic | A | 1,828,308 | 2173 |
| BSB1322 | Chronic | A | 1,796,313 | 2173 |
| BSB1324 | Chronic | A | 1,122,264 | 2302 |
| BSB1331 | Chronic | A | 1,369,995 | 2183 |
| BSB1364 | Occult | E/A | 686,958 | 2156 |
| Average |  |  | 1,063,988 | 2208 |

^1^ HIV co-infected with HIV RNA = 4,075 copies/mL

^2^ HIV co-infected with HIV RNA = 80,170 copies/mL
